# Color Stability of Glass Ionomer Cement after Reinforced with Two Different Nanoparticles

**DOI:** 10.1155/2020/7808535

**Published:** 2020-05-31

**Authors:** Sharat Chandra Pani, Moath Tofik Aljammaz, Abdullah Mohammed Alrugi, Abdulaziz Mohammed Aljumaah, Yazeed Minahi Alkahtani, Abdulaziz AlKhuraif

**Affiliations:** ^1^Sharat Chandra Pani Schulich School of Medicine and Dentistry, Western University, London ON, Canada; ^2^College of Dentistry, Riyadh Elm University, Riyadh, Saudi Arabia; ^3^College of Allied Medical Sciences, King Saud Univeristy, Riyadh, Saudi Arabia

## Abstract

**Aim:**

This study aimed to compare the staining characteristics of a commercially available restorative glass ionomer cement to a formulation reinforced by the addition of carbon nanotubes and another formulation reinforced by the addition of silver nanoparticles to the powder of the same cement. *Methodology*. Twenty samples each of a control glass ionomer cement (PULPDENT® Glass Fill®, Pulpdent Corp. Watertown, MA, USA), control cement reinforced with 0.0006 gm (0.03% by weight) of carbon nanotubes (Sigma Aldrich, St Louis MO, USA), and control cement reinforced with 0.2 gm (10% by weight) of silver nanoparticles (Nanocyl™, Nanocyl SA, Sambreville, Belgium) were immersed in a staining solution. Color evaluations were carried out after 1 h, 24 h, and 1 week. Color change values were calculated.

**Results:**

The results indicated that carbon nanotube reinforced specimens exhibited less color stability when compared to controlled glass ionomer cement specimens; however, both samples had significantly greater color stability than silver nanoparticle reinforced glass ionomer samples.

**Conclusion:**

It can be concluded within the limitations of this study that carbon nanotube reinforced glass ionomer cements have better color stability than silver nanoparticle reinforced glass ionomer cements.

## 1. Introduction

Glass ionomer cements were first developed by Wilson and Kent in the 1960s [1]. Their high fluoride release and chemical bonding to the tooth structure make them the cement of choice for several restorative and luting functions [1]. However, their high solubility and relatively low compressive strength have meant that since the 1980s, researchers have sought to improve the strength of glass ionomers [1, 2].

The earliest attempts on creating reinforced cements focused on combining the glass powder with readily available materials such as sliver alloy from dental amalgam [2]. However, this was soon replaced with the sintering of specific metal alloys from manufacturers [3, 4]. The advent of nanotechnology at the turn of the 21^st^ century brought about new scope for the reinforcement of glass ionomer cements [5]. Over the past decade, glass ionomer powder was reinforced with different nanoparticles, ranging from amorphous materials such as hydroxyapatite to metals [3–5].

The chemical structure of the glass ionomer is based on the formation of a gel matrix [1]. To this extent, it has been hypothesized that the addition of fibrous materials would reinforce this structure [3, 4, 6–8]. Carbon nanotubes are available in the form of fibers and have been shown to greatly improve the structure and nature of gel matrices [8].

Though glass ionomers lack the esthetic effects of composites, they are often used as an esthetic restorative material, especially in primary teeth [1]. It has been shown that the use of additives reduces the color stability of glass ionomers [9]. However, the extent and clinical significance of this color instability varies depending on the type of filler particle used. Of the several methods that can be used to detect color changes, it has been demonstrated that the use of the *L* *∗* *a* *∗* *b* axis using the CIELAB system is the most preferred [10–12]. The system allows for the use of reliable, quantifiable, and reproducible recording of color changes.

Several authors have compared the staining potential of different substances such as coffee, tea, or aerated beverages on the teeth or restorative materials. It has been demonstrated that in order to compare staining characteristics of different materials, it is preferable to use a customized staining solution [13, 14].

The present study aimed to compare the staining characteristics of a commercially available restorative glass ionomer cement to those of customized powders prepared by adding carbon nanotubes to glass ionomer cement powder or silver nanoparticles.

## 2. Materials and Methods

Ethical approval was obtained, and the study was registered in the research center of the Riyadh Elm University (FUGRP/2018/146).

Sample power calculation was done using the GPower sample power calculator (Universitat Kiel, Kiel, Germany). It was estimated that in order to achieve a sample power of 0.95 (95% confidence interval) and effect size of 0.8, each group would have to comprise 20 samples.

### 2.1. Preparation of the Samples

The powder of the cements used in this study comprised one of the three following groups:

- Control group (*n* = 20): commercially available restorative glass ionomer cement (PULPDENT® Glass Fill®, Pulpdent Corp. Watertown, MA, USA) was used in the study.
- Carbon nanotubes (*n* = 20): 2 gm of restorative glass ionomer powder cement (PULPDENT® Glass Fill®, Pulpdent Corp.) was reinforced with 0.0006 gm (0.03% by weight) of carbon nanotubes (Sigma Aldrich, St Louis MO, USA).
- Silver nanoparticles (*n* = 20): 2 gm of restorative glass ionomer powder cement (PULPDENT® Glass Fill®, Pulp Dent Corp) was reinforced with 0.2 gm (10% by weight) of silver nanoparticles (Nanocyl™, Nanocyl SA, Sambreville, Belgium).

All samples were mixed using the polyacrylic acid-based liquid provided by the manufacturer cement (PULPDENT® Glass Fill®) as per the manufacturer's instructions and a powder liquid ratio of 3 g of powder for 1.5 ml of liquid. The prepared mixtures were poured into a silicone mold of diameter 10 mm with a height of 2 mm. A total of 15 tablets per group were prepared for analysis.

One liter of a standard staining solution was prepared by mixing coffee, cola, and cranberry juice in equal proportions using a methodology previously described [14]. The tablets were immersed completely in the solution, and measurements of color were performed using a previously developed protocol [13].

Color evaluations were carried out after 1 h, 24 h, and 1 week.

After rehydration, the samples were rinsed and dried with filter paper, and the baseline color measurements were performed using a spectrophotometer (Gretag Macbeth Color-Eye® 7000A). Color evaluations were made with color parameters based on average daylight (D65: 6504 K) and illuminating view geometry *d*/10. Calibration was made using a white standard. Individual specimens were placed on aperture, and readings were recorded according to Commission Internationale de l'Eclairage *L* *∗* *a* *∗* *b* *∗* color space (CIELAB). The overall change in color (Δ*E*) values was calculated at each interval using the formula

Δ*E* = [(*L* *∗* 1 − *L* *∗* 2)^2^ + (*a* *∗* 1 − *a* *∗* 2)^2^ + (*b* *∗* 1 − *b* *∗* 2)^2^]^1/2^ based on the readings obtained on the CIELAB scale. The Δ*E* values were calculated from baseline to one hour, one hour to one day, and one day to one week, respectively.

### 2.2. Statistical Analysis

The color changes between groups at each time interval were compared using the one-way ANOVA with Scheffe's post hoc test. The change in color for each sample from interval to interval was evaluated using the repeated measures ANOVA.

## 3. Results

When the delta *E* values from baseline to the end of the first hour were compared, it was seen that the test groups had lower values when compared to the two test groups. The one-way ANOVA found this difference to be significant (Table 1). The Scheffe's post hoc test showed that while the control group showed significantly less color change when compared to test groups, the carbon nanotubes reinforced GIC had significantly lower color change than the silver nanoparticles reinforced GIC (*p* < 0.05).

At the end of one hour, it was observed that there were significant differences between the groups. The post hoc test (Table 2) showed that the control group had significantly lower color change than the test groups. Among the test groups, the carbon nanotubes reinforced GI had a significantly lower color change than the silver nanoparticles reinforced GI. At 24 hours (Table 3), although the control group had the lowest color change, there was no significant difference between the control group and the carbon nanotube reinforced glass ionomer (*p*=0.238). The silver nanoparticle reinforced GI had a significantly higher color change than the other two groups (*p* < 0.05). At 7 days (Table 4), the control group had significantly lower color change than the test groups. Among the test groups, the carbon nanotube reinforced GI had a significantly lower color change than the silver reinforced GI.

The repeated measures ANOVA (Table 5) showed that while there was a significant difference in color change across the tested groups, there was no significant change in the pattern of color change observed (*p*=0.339).

## 4. Discussion

The use of the CIELAB for the evaluation of tooth colored restorative materials is considered a universally acceptable technique [15].

The results at one hour showed significant differences between the three groups suggesting that reinforcing glass ionomer cement with any particle reduces color stability. However, a closer examination of the data reveals the vast difference between the glass ionomer reinforced with carbon nanoparticles and that reinforced with silver nanoparticles. Data from 24 hours to 7 days emphasize this point showing that silver nanoparticles had significantly lower color stability than the control group, while the carbon reinforced cement had no significant differences when compared to the control. This fact may be of interest to researchers given the large volume of data emerging on the potential mechanical superiority of glass ionomers reinforced with silver nanoparticles [16, 17].

The use of the *L* *∗* *a* *∗* *b* color axes for the measurement of color change is universally accepted as a better method than using more subjective shade guides [18, 19]. However, the accuracy of the system viewed keeping in mind that while values for Δ*E*^*∗*^ between 1 and 3.3 can be detected by some observers in standardized conditions, a Δ*E*^*∗*^ value below 1 is generally considered imperceptible even to a trained eye [18]. All the materials used in this study displayed clinically perceptible color change, and the difference is color stability between the groups at each measurement interval which was above the clinical threshold of Δ*E*^*∗*^ = 1.

The color stability values in this study must be viewed keeping in mind both esthetic and structural considerations. The use of sliver and carbon nanoparticles to increase the strength of glass ionomer cements has been previously addressed in the literature [16, 20]. It has been shown that the increase in mechanical properties brought about by the addition of even minor amounts of carbon nanoparticles justifies research into the properties of such a mixture [20]. In the case of silver nanoparticles, in addition to the increase in physical properties, their addition to glass ionomer cements also enhances the antimicrobial properties of these cements [16, 17, 20]. However, the color stability offered by these reinforced glass ionomer cements may not, in their current form, be adequate for their use as anterior esthetic restorations.

The relationship between the nature of the reinforcing particle, the powder liquid ratio, and solubility of the glass ionomer cement has been extensively documented in the literature [2, 3, 6, 21]. One of the suggested advantages of using nanoparticles is that, given their low size, it is possible to maintain suggested powder liquid ratios and still achieve a sustainable aluminofluorosilicate gel matrix [6]. In our current study, while we used the same powder liquid ratio by weight, it is clear that the cement reinforced with silver nanoparticles showed significantly lower color stability than the cement reinforced with carbon nanofibers. One possible explanation for this is that the volume of fibers per gram greatly exceeds the volume of a similar weight of silver nanoparticles. This seems to support the argument for the use of fibril-based nanoparticles [4, 7, 8]. However, the details of the merits of each type of particle are beyond the scope of this study.

The results of this study must be viewed keeping in mind certain limitations. The greatest limitation is the baseline shade acceptability of the carbon nanotube reinforced glass ionomer [20]. While the carbon nanotube reinforced glass ionomer has better color stability than the glass ionomer reinforced with silver nanoparticles, more research is needed to establish ways to improve the baseline esthetic acceptability of carbon nanotube reinforced glass ionomer cement. Further research into the nature of nanofibers as compared to spherical nanoparticles will also help future researchers develop stronger more esthetically acceptable glass ionomer cements.

## 5. Conclusions

Based on the methodology employed and the results obtained, it may be concluded that:

- The addition of reinforcing materials significantly reduces the color stability of glass ionomer cement.
- The addition of silver nanoparticles creates significantly lower color stability than the addition of carbon nanotubes.

## Figures and Tables

**Table 1:** Overall color change patterns (Δ*E*) for the different groups.

		*N*	Mean Δ*E*	Std. deviation	Std. error	95% confidence interval for mean		*F* ^*∗*^	Sig.
						Lower bound	Upper bound		
<0.001 hour	Control	15	1.3460	0.66854	0.17262	0.9758	1.7162	22.434	<0.001^*∗∗*^
	Silver nanoparticle reinforced	15	22.1340	3.30876	0.85432	20.3017	23.9663		
	Carbon nanotube reinforced	15	5.8660	1.99908	0.51616	4.7589	6.9731		

24 hours	Control	15	2.7567	1.22313	0.31581	2.0793	3.4340	19.491	<0.001^*∗∗*^
	Silver nanoparticle reinforced	15	18.4353	3.63610	0.93884	16.4217	20.4489		
	Carbon nanotube reinforced	15	4.1033	0.92527	0.23890	3.5909	4.6157		

7 days	Control	15	3.3753	1.09621	0.28304	2.7683	3.9824	18.677	<0.001^*∗∗*^
	Silver nanoparticle reinforced	15	23.9000	4.31245	1.11347	21.5118	26.2882		
	Carbon nanotube reinforced	15	9.7593	5.92823	1.53066	6.4764	13.0423		

^*∗*^Calculated using one-way ANOVA.^*∗∗*^Differences significant at *p* < 0.05.

**Table 2:** Difference in color change among different groups at one hour.

Scheffe^a^ group	*N*	Subset for alpha = 0.05		
		1	2	3
Control	15	1.3460		
Carbon nanotube reinforced	15		5.8660	
Silver nanoparticle reinforced	15			22.1340
Sig.		1.000	1.000	1.000

Means for groups in homogeneous subsets are displayed. ^a^Uses harmonic mean sample size = 15.000.

**Table 3:** Difference in color change among different groups at 24 hours.

Group	*N*	Subset for alpha = 0.05	
		1	2
Control	15	2.7567	
Carbon nanotube reinforced	15	4.1033	
Silver nanoparticle reinforced	15		18.4353
Sig.		0.281	1.000

Means for groups in homogeneous subsets are displayed. ^a^Uses harmonic mean sample size = 15.000.

**Table 4:** Difference in color change among the different groups at 7 days.

Scheffe^a^ group	*N*	Subset for alpha = 0.05		
		1	2	3
Control	15	3.3753		
Carbon nanotube reinforced	15		9.7593	
Silver nanoparticle reinforced	15			23.9000
Sig.		1.000	1.000	1.000

Means for groups in homogeneous subsets are displayed. ^a^Uses harmonic mean sample size = 15.000.

**Table 5:** Repeated measures test for significance of pattern of color change among groups.

Source	Type III sum of squares	d*f*	Mean square	*F*	Sig.
Intercept	490.022	1	490.022	3.548	0.066
Group	129.067	1	129.067	0.935	0.339
Error	5938.561	43	138.106		

## Data Availability

The data used to support the findings of this study are available from the corresponding author upon request.
